# A bioethical framework to guide the decision-making process in the care of seriously ill patients

**DOI:** 10.1186/s12910-018-0317-y

**Published:** 2018-08-20

**Authors:** Daniel Neves Forte, Fernando Kawai, Cláudio Cohen

**Affiliations:** 1 Palliative Care Program, Hospital Sírio-Libanês, R. d. Adma Jafet, São Paulo, SP CEP01308050 Brazil; 2 Hospice and Palliative Medicine Fellowship, New York Presbyterian Queens, Clinical Medicine, Weill Medical College of Cornell University, New York, NY USA; 3 Department of Legal Medicine and Medical Ethics, Medicine School, University of São Paulo, São Paulo, Brazil; 4 Post-doctoral fellow on Bioethics, Medicine Shool, University of São Paulo, São Paulo, Brazil

**Keywords:** Decision-making, Bioethics, Evidence-based practice, Person-centered care

## Abstract

**Background:**

One of the biggest challenges of practicing medicine in the age of informational technology is how to conciliate the overwhelming amount of medical-scientific information with the multiple patients’ values of modern pluralistic societies. To organize and optimize the the Decision-Making Process (DMP) of seriously ill patient care, we present a framework to be used by Healthcare Providers. The objective is to align Bioethics, Evidence-based Practice and Person-centered Care.

**Main body:**

The framework divides the DMP into four steps, each with a different but complementary focus, goal and ethical principle. Step 1 focuses exclusively on the disease, having accuracy is its ethical principle. It aims at an accurate and probabilistic estimation of prognosis, absolute risk reduction, relative risk reduction and treatments’ burdens. Step 2 focuses on the person, using empathic communication to learn about patient values and what suffering means for the patient. Emphasis is given to learning and active listening, not taking action. Thus, instead beneficence, we trust comprehension and understanding with the suffering of others and respect for others as autonomous moral agents as the ethical principles of Step 2. Step 3 focuses on the healthcare team, having the ethics of situational awareness guiding this step. The goal is, through effective teamwork, to contextualize and link rates and probabilities related to the disease to the learned patient’s values, presenting a summary of which treatments the team considers as acceptable, recommended, potentially inappropriate and futile. Finally, Step 4 focuses on provider-patient relationship, seeking shared Goals of Care (GOC), for the best and worst scenario. Through an ethics of deliberation, it aims for a consensus that could ensure that the patient’s values will be respected as well as a scientifically acceptable medical practice will be provided. In summary: accuracy, comprehension, understanding, situational awareness and deliberation would be the ethical principles guiding each step.

**Conclusion:**

Hopefully, by highlighting and naming the different perspectives of knowledge needed in clinical practice, this framework will be valuable as a practical and educational tool, guiding modern medical professionals through the many challenges of providing high quality person-centered care that is both ethical and evidence based.

## Background

What is the right thing for providers to do when caring for patients facing a life-threatening illness? In the last 2 decades we’ve witnessed an exponential growth of this answer’s complexity. The big data era adds an overwhelming amount of medical-scientific publications, puzzling the clinician’s capacity to appreciate risks and benefits of treatments. Meanwhile, today’s multicultural societies require new skills from providers to learn and respect the diversity of patient values [1]. Considering the increasing time pressure, it’s not surprising that medical decisions are evermore contentious [2, 3].

As a way to protect themselves and promote autonomy, clinicians sometimes avoid the responsibility of decision-making, and delegate it to patients and surrogates. Such “solitary” autonomy, nevertheless, might inadvertently undermine patient care by depriving patients of the professional guidance needed to make decisions, especially in critical situations [4–6]. We believe that the principlism model, based on beneficence, non-maleficence, autonomy and justice, may foster this trend. Facing such controversies at the bedside, physicians are sometimes caught in false dichotomies, as if the solution could be presented in only two excluding answers: autonomy or beneficence. This happens for example in a frequent modern conflict: the disagreement between the Intensive Care Unit (ICU) physician and the oncologist regarding the ICU admission of an advanced cancer patient with an acute deterioration. The intensivist, possibly focused on the high mortality, can disagree with the admission. Meanwhile, the oncologist, possibly concerned about the patient’s fear of death, recommends it. Contemporary ethical reasoning may approach this situation as an autonomy vs. beneficence/maleficence dilemma, where one principle may prevail. Alternatively, providers could even refrain from this difficult discussion and delegate the decision to the patient/surrogate, sometimes by simply asking “what would you like for us to do?” despite the patient/surrogate’s lack of knowledge about the risks and benefits involving each choice. Such ethical dilemmas may arise in other contexts and other diseases, such as Emergency Room or ambulatory settings, involving for instance decisions about whether to start a new course of antibiotics on a patient with advanced dementia or whether to implant a left-ventricular assisted device in a patient with advanced heart failure, among others difficult decisions. We will use the oncologist vs. intensivist dilemma to give a practical perspective of the framework. However, the same framework could also be used in different settings and diseases as well.

To address these challenges, we propose a practical framework to guide the Decision Making Process (DMP) during the care of a seriously ill patient, aiming to align Evidence-Based Practice (EBP) and Person-Centered Care using a bioethical referential.

## Main text

### The framework

The framework divides the DMP in four steps, each one with a different but complementary goal and ethical principle, as summarized in Table 1.

**Table 1:** Summary of the Decision-making framework

Step# and guiding ethical principle	Main focus	Goals	Method	Practical objectives
1st step: Ethics of Accuracy	The body and its biology: the diseases and treatment options	To be accurate in diagnosis, prognosis, and success and failure rates of possible treatments.	Evidence-based practice, probabilistic and scientific reasoning	To Present: a) A correct diagnosis and the phase of the disease. b) A probable prognosis, i.e., how the majority of patients with that phase of the disease will evolve over time. c) The absolute risk reduction, the relative risk reduction, the number needed to treat. The potential adverse effects as well as costs and inconveniences of treatments. Uncertainty is addressed through estimation of confidence intervals and the disclosure of the potential limitations of generalization of evidence in that specific case.
2nd step: Ethics of comprehension	The person and biography: patient’s values and views of suffering.	To comprehend and be empathetic to the patient’s suffering, respecting the other as an end in itself. The emphasis here is to listen, to be present and to learn about biography, not to act, to change or to fix it.	Empathic communication	To learn about the patient’s understanding, views, life values, perceptions of suffering and treatment preferences.
3rd step: Ethics of situational awareness	The healthcare multidisciplinary team	To apply the scientific evidence developed in populations to the specific situation of the patient, conciliating evidence based treatments with the patient’s values and biography.	Clinical judgement and effective team communication	A team proposal of which would be considered: a) the acceptable evidence based treatment, taking on consideration the scientific view only b) The best or recommended treatment following both the scientific evidence while also respecting the patient’s biography and wishes. c) The potentially inappropriate treatments following scientific evidence and the patient’s biography and wishes. d) The futile or unacceptable treatments, considering exclusively the scientific basis.
4th step: an ethics of deliberation	The patient-provider relationship	Establishing rapport and building consensual patient-provider goals of care, ensuring that the patient’s values will be respected and scientifically acceptable practices will be used.	Deliberation and person-centered communication	To have and honest and empathic communication about diagnosis, prognosis and then, to establish consensual goals of care for the best and worst scenario. After the setting of GOC, specific treatments within the Step#3 proposal are deliberated between patient and physician, reaching a new consensus about which treatments might or might not be employed to reach the desired goals.

In practice, the differences between these steps are often unclear and may prompt miscommunication and conflicts. By separating and naming the goal of each step, the framework may foster changing the negotiation focus from positions (to admit or not, to prioritize autonomy or beneficence) to focus on interests [7], potentially reaching a better decision. For example, if an oncologist has a longer relationship with the patient, he may have an optimistic bias about prognosis [8]. The Framework’s first step discusses exclusively the disease, aiming only at accurate probalisitic predictions. In a second step, the intensivist could use comprehension, and learn about the patient’s biography and values. And then, considering EBP and the patient’s values, providers could discuss which treatments would be considered acceptable, which they would recommend, which might be potentially inappropriate and which would be scientifically unacceptable. After expanding the options beyond the yes/no stage, they could involve the patient/surrogate in the DMP towards a consensual goal of care, for example “to attempt to prolong life even if it involves suffering and poor quality of life”, or “to survive, as long as not bed-bound” or “to allow nature to take its course and avoid unnecessary suffering”. Depending on the agreed goal, options could include for example: 1) Full Code ICU admission, or 2) ICU trial of 3–5 days, then reassess and consider Do Not Ressucitate (DNR)/ comfort care if clinical deterioration or 3) DNR/ Do Not Intubate (DNI), comfort care only and no ICU.

The Framework may potentially help to organize the reasoning process. The fundaments of each step are different, but complementary.

### First step: Ethics of accuracy, focusing on diseases

Clearly stating this first step of focusing on body and disease can help clinicians to improve accuracy. At the same time, it helps physicians to remember that this is only the *first* step with more to come.

That being said, despite the medical-scientific development, being accurate in the twenty-first century has become very challenging. The amount of noise in the big data era poses a threat to accuracy. The amount of medical-scientific publications with the word “scientific” has grown from an average of 510 new articles/ year in the early 1900s (personal PUBMED search, Excel file) to an average of 18,703 articles/ year in the first six years of the 2010’s. Anyone wanting to read all the articles on cancer published in 2014 would need to read 400 articles/ day. As Nate Silver puts it, although the amount of information is increasing with amazing speed, the same cannot be said for the amount of useful information. Most of it is just noise, which is increasing faster than the signal [9].

This overwhelming amount of information may cause cognitive discomfort, increasing our trend to a heuristic confirmation, resulting in inaccurate predictions [10]. In a recent systematic review, clinicians more often underestimated rather than overestimated harm, while overestimated rather than underestimated benefits [11]. More than 100 biases and heuristics affecting clinical decision-making have been described [12]. All this noise, heuristics and cognitive bias may contribute to an approach excessively focused on curative or life-prolonging treatment for seriously ill patients even in the last weeks of life [13]. Base rate neglect is possibly the most relevant heuristic threatining accurate predicitons [14]. It happens when details of the narrative lead to making inferences that violate norms of probabilistic reasoning. For example, a narrative of a patient with terminal lung cancer who wishes to survive to care for her children may lead to an excessively optimistic prediction, posing a threat to accuracy, despite its benevolent intention. The discomfort between the cognitions “I think she’ll die soon” and “I don’t want her to die” may be alleviated with the cognition of an overestimated, but inaccurate, prognosis prediction. And this may happen due to base rate neglect- in such case, the base rate may be estimated thorugh the Palliative Performance Index, which would provide the information that most patients in that situation would die within days.

By initially focusing exclusively on the disease and aiming at an accurate prediction in this step, we may separate predictions from desires, reduce cognitive dissonance, minimize bias, and increase accuracy [14, 15]. A classic Kahneman’s method to overcome bias would start with the prediction of the base rate, i.e., first ignoring all the biographical and specific information, prioritizing a macro estimation, i.e., the base rate. Base rate could be prevalence or expected mortality of all cases with similar presentation. Today’s medical scoring systems, based on the analysis of thousands of patients, can offer this information [16]. After this initial estimation, case peculiarities are then used to refine the estimation. In accurate predictions, the order of these events does matter. Keeping it in terms of probability would help physicians to refrain from deterministic thinking (yes/no), developing instead probabilistic reasoning [15].

Probability underlines that prognostication is not a foretelling prophecy but rather it is a factor in risk prediction. Uncertainty is not only admitted as inherent, but openly discussed and estimated [17].

And to make accurate predictions, we have to leave the comfort of confirmation bias and move towards an effort to contemplate disconfirmation. “Why is our hypothesis wrong?” is a more important question than “Why is our hypothesis correct?” [10, 14, 15]. Providers could focus on estimation of prognosis, rates of absolute risk reduction, relative risk reduction, number needed to treat, potential adverse effects and costs and burdens of treatments. Uncertainty might be addressed through estimation of confidence intervals and the disclosure of the potential limitations of generalization of evidence in that specific case.

Thus, the first step is primarily governed by an ethics of accuracy and uses as main tools EBP and probabilistic reasoning.

### Second step: Ethics of comprehension and understanding, focusing on the person

An accurate decision about the disease is necessary, but a decision about the disease alone is not enough to define a right decision. A right decision should also include the relief of suffering. However, to do so, the provider should learn about what is suffering for the patient. This requires an understanding attitude, an effort to tolerate listening to the suffering shared by the patient without trying immediately to fix it. Moreover, it requires a method that is not included in natural sciences.

Knowledge of Natural sciences, such as physics or biology, requires an objective knowledge, measurement of its variables and analysis of its relantionships within a theory, using mathematical relationships to explain its efficient causes. Modern natural sciences exclude intentionality and anthropomorphism, substituting the teleological search of “why” for the efficient search of “how”. Moreover, a natural science theory has to be refutable and generalizable. On the other hand, human suffering has a subjective nature, impregnated with cultural meanings, affections and memories, making it difficult to be measured or refutable. Its singular, idiosyncratic nature, cannot be generalized. Suffering is experienced by people, not merely bodies, and has its sources in challenges that threaten the wholeness of individuals as complex social and psychological entities [18]. .And since human suffering is dressed with language, excluding its intentionality would exclude a fundamental part of its nature.

Instead of using natural science to learn about suffering, a human being can use empathic communication to learn about another another human being’s suffering. In clinical practice, natural science methods can provide accurate predictions about the natural course of lung cancer as well as the efficacy of its treatments. However, to reach a right decision, providers should not ignore the influence of the fear of death, the social isolation and financial stress related to loss of functional status and possible spiritual distress that can be manifested in questions such as “What is the meaning of this cancer?”

Thus, the second step explicitly emphatizes the learning about a patient’s biography and values. Values could be defined as “the collection of goals, expectations, predispositions and beliefs that individuals have for certain decisions and their potential outcomes” [19]. Values are meanings amidst a biographical construct, where several narratives take place through a dialectical process. To learn about values and suffering, a provider has to consider wider aspects of the language, for instance, its pitch, accent, rhythm, intonation, called its prosody [20]. Differences in prosody can give almost opposite meanings to the same phrase. “Do you have a problem?” can mean a threat or an offer to help, depending on the prosody. The non-verbal information, the connotation and the socio-cultural referential should be considered as well, since they give meaning to the cognitive and affective aspects from which values are derived.

To learn about patient values, we suggest some guiding questions, asked with empathetic communication techniques adapted both to the providers’ and patient’s coping styles, (Table 2). In this step, emphasis is given to learning and active listening, not acting to find a solution or solve a problem. The challenge for the providers in this step is to be understanding of the patient, to listen and learn about his/ her suffering, with a more contemplative attitude. Perhaps the ethical principle of beneficence (a term which connotes acts of mercy, kindness, and charity) [21], and historically, the ethical basis of the paternalistic medical acts, would not be appropriate. Thus, we trust comprehension and empathy towards the suffering of others and respect for others as autonomous moral agents as the ethical principles of this step.

**Table 2:** Domains to be explored to understand the patient as a person^a^

#	Domain	Examples of questions
1	Identification	How do you prefer to be called? Where do you come from? Profession? Married? Children?
2	Surrogate	If you were not able to make decisions, with whom would you want physicians to discuss your medical condition? Who would you want to make decisions for you?
3	Preferences about receiving medical information	What are your preferences about receiving medical information? If we had bad news, would you want to know about it or should we discuss it just with your family? Are you the kind of patient who appreciates knowing all the details about your disease, or do you prefer to know just the big picture and that I talk about the details with someone else?
4	Preferences for participation in medical decisions	What are your preferences for participation in medical decisions that may possibly involve life threatening situations or risk of permanent disability?
5	Relevant values and view of suffering	What is important for you? How is your life outside the hospital? What are you hoping for? What are your biggest concerns right now? What is the hardest part of being ill for you? And for your family? Given what we are facing, what is your main goal? (for patients who answer “to be cured”, after acknowledging hope, healthcare providers can ask “what else?”). Other relevant values that could be explored: values about maintenance of bodily integrity/ physical well-being/functional status/ independence / cognitive function /autonomy and independence / social and emotional engagement/ avoiding burdensome physical symptoms. Spirituality might be learned by using acronyms for obtaining a spiritual history. For example, FICA or SPIRIT: FICA (Puchalski20) F: Faith and beliefs I: Importance of spirituality in the patient’s life C: Spiritual community of support A: Addressing the patient’s spiritual issues in his or her care SPIRIT (Maugans21) S: Spiritual belief system P: Personal spirituality I: Integration with a spiritual community R: Ritualized practices and restrictions I: Implications for medical care T: Terminal events planning
6	End of Life preferences	If facing a terminal and irreversible illness, how would you like to be cared for? Would you prefer to have your life prolonged even if that could involve suffering and no quality of life? Or should we try to attempt to prolong life as long as some functional independence is possible? Or should we let nature take its course, focusing just on the relief of suffering? Should we solely focus on minimizing suffering and pain, even if it eventually may hasten death?
7	Allowing space for comments and clarification of doubts	Did I forget to ask something important? Would you like to tell me anything else? What did you think about this conversation?

^a^ adapted from: Back et al. [28], Scheunemann et al. [31], Curtis et al. [26], Bernacki et al. [25] and Sulmasy D [32].

### Third step: Ethics of situational awareness, focusing on providers

Third step focuses on improving the clinical judgement of the healthcare team. Clinical judgement can be defined as the exercise of reasoning under uncertainty when caring for patients [22]. It combines Step#1, Step#2 and the context where the stuation is happening. The challenge here is to improve judgement through improvement of situational awareness. The goal is to try to overcome practice based on reflex and adopt practice based on reflection and teamwork.

Situational awareness involves being aware of what is happening in the vicinity to understand how information, events, and one’s own actions will impact goals and objectives, both immediately and in the near future [23, 24]. Specifically, using teamwork communication, providers should contextualize the 1st and 2nd steps, linking rates and probabilities related to the disease to the learned patient’s values. For example, how can we contextualize the knowledge about the most accurate prognostic probabilities regarding a potentially fatal disease and its treatments with the most comprehensive knowledge about the patient’s value on being independent and his/her fears about physical suffering and social isolation? Which decisions would provide the best cost-benefit balance, including the time spent in the treatment? Reflecting upon these questions is central either to EBP or Person-Centered Care. And overconfidence, i.e., a mismatch between perceived and actual performance, may threaten such reflection and decrease teamwork [15].

Nonetheless, improving situational awareness through effective teamwork and feedback can reduce overconfidence [24]. The cognitive aspect of awareness (i.e., “not knowing what you don’t know”) is situation specific. Rarely, the reason for not knowing may be from a lack of knowledge per se, such as seeing a patient with a disease that the physician hasn’t encountered before [10]. More commonly, cognitive errors reflect problems in gathering data, failure to recognize the significance of data or failure to “put it all together.” The cognitive component also includes a failure of metacognition (the willingness and ability to reflect on one’s own thinking processes and to critically examine one’s own assumptions, beliefs, and conclusions). Therefore, teamwork and feedback seeking behavior may reduce overconfidence. It can be fostered by behaviors and attitudes, such as [23]:

- Using and encouraging self-disclosure, sharing thoughts and feelings related to the situation
- Practicing and encouraging active listening and openness to feedback from different team members
- Using assertiveness and constructive criticism, focusing on the team’s best interests rather than individual views only
- Through effective teamwork, the main goal is to apply the population based scientific evidence while also adapting it to the specific situation of the patient, including the patient’s biology, biography and cultural aspects.
  Moreover, the judgements can be expanded beyond an “all or nothing” model. Through discussions, the team could reach a summary of what the team considers to be:

- The acceptable evidence based treatment, taking into consideration only the scientific view
- The best or recommended treatment following both scientific evidence while also respecting the patient’s biography and wishes.
- The potentially inappropriate treatments following scientific evidence and the patient’s biography and wishes.
- The futile or unacceptable treatments, considering exclusively the scientific basis.

Such approach could broaden the discussion, creating new options by fostering possible solutions considered acceptable. At the same time, it could protect patients and the healthcare system from overtreatment practices resulting from overconfident judgments [24]. For example, in the case of a patient with metastatic lung cancer with septic shock and acute respiratory failure, the team could recommend a) ICU admission for Sepsis treatment and intubation; b) Limited trial of ICU treatment and intubation but transition to comfort care only if no significant improvement in order to respect the patient’s wishes to avoid prolongation of pain and unnecessary suffering; c) recommendation against prolonged ICU admission and tracheostomy (in order to avoid prolonged pain) and d) recommendation for a DNR order since Cardiopulmonary Ressucitation (CPR) would very unlikely offer any benefit in the setting of metastatic cancer.

### Fourth step: Ethics of deliberation, focusing on the patient-provider relationship

Last step would focus on patient-provider relationship, seeking shared goals of care (GOC), aligning a medically appropriate treatment with the patient’s valued goals [25]. We consider that GOC should fall within a spectrum between two extremes. At one extreme to maximize any chance of extending life while minimizing unnecessary suffering, and at the other to maximize the relief of suffering while not attempting to artificially prolong life. In between these two extremes, lies a grey area, where the goal could be, for instance, to prolong life as long as no more painful interventions are added. To establish the appropriate GOC, the team would attempt to conciliate the patient’s values and the risks and benefits of interventions. In situations with both a very poor prognosis and a high level of certainty, clinicians could provide an informed assessment and suggest a plan based on the patient’s values [26]. For example, providers could recommend a comfort care approach for the surrogate of an unconscious advanced cancer patient, whose prior known wishes were “not to suffer”. If the surrogate agrees, the team could then place a DNR order. Or, similarly, clinicians can make an informed assent in favor of treatment when there is a very good prognosis and a very high level of certainty (for instance, in a patient with a large pleural effusion the team could recommend in favor of thoracentesis to minimize dyspnea). However, in most real life scenarios, there are many uncertainties about risks and benefits of treatments, also with uncertainties about their impact on the patient’s core values, so the decision about goals of care would require a deliberative dialogue.

Deliberation about GOC is wider than simply asking patients about “what they want” or “if they want to go to the ICU”. Since patients do not necessarily have full understanding to answer such questions, simply asking does not necessarily guarantee autonomy. Within a medical context, decision capacity refers to the abilities needed to utilize information regarding a proposed treatment option to make a congruent choice with one’s own values. It comprehends understanding, expressing a choice, appreciation, and reasoning [27]. Notwithstanding, deliberation about GOC is broader than simply asking what does someone wants. Best practices in discussing GOC include sharing prognostic information, eliciting decision-making preferences, understanding fears and goals, exploring views on trade-offs and impaired function, as well as wishes for family involvement [25, 26]. It is required to empathically inform the patient/surrogate about his/her situation, answer questions, clarify any doubts and acknowledge emotions, accepting that the patient/surrogate requires time to process and “digest” this very difficult information [28, 29]. Moreover, as highlighted since Aristotle, more important than discussing *means* (in our times, for example, ICU admission) is to discuss *ends* (for example, try to prolong life regardless of its quality).

The quest for consensus building should guide Step 4. And whenever an effective, efficient and safe medical intervention harms a patient’s core values, causing suffering regarded as intolerable and pointless by a capable patient, we believe that providers should prioritize these values. After all, the ethical imperative of treating a person as a whole is more important than treating just the illness. When no consensus can be reached, the decision could be escalated to the institutional level so that a third party can intermediate the deliberation process. In view of the evidence, consulting a Bioethics Committee might be the best initial step for these situations [30].

## Conclusion

This framework could be valuable as a practical and educational tool, guiding modern medical professionals through the many challenges of providing high quality person-centered care that is both ethical and evidence based. It has, however, some limitations. First, the framework has not been validated. It is a conceptual model, based on practice, reflection and expert opinion. Nevertheless, the DMP based on this framework could be tested, to refute our hypothesis or not. Secondly, it could be argued that there is no new information in each step. However, we believe the main strength of this framework is to link several areas of knowledge into a single process, acknowledging the limits and aims of each kind of epistemology forming a practical and didactical tool. Moreover, its steps could be used at separate times or be applied by different professionals. For instance, the first step could be applied by a field expert, or even by a machine with proper input, such as IBM Watson®, generating a standardized report. The second step could be applied by any professional with the ability to communicate with empathy and compassion. Possibly, the third and fourth steps are within the core of the medical profession, and likely couldn’t be replaced by machines or other professionals. Perhaps, the use of DMP frameworks such as the one presented here could aid in maximizing the efficacy and efficiency of the healthcare practice. We understand that this will not be easy nor simple, but, hopefully, it will improve access to healthcare with high quality and ethical standards.

## Abbreviations

CPR: Cardiopulmonary Ressucitation
DMP: Decision-Making Process
DNI: Do Not Intubate
DNR: Do Not Ressucitate
EBP: Evidence-based practice
GOC: Goals of care
ICU: Intensive Care Unit
